# Initial Screening of Extrachromosomal Circular DNA Candidates for Pork Meat Quality Traits Using Circle-Seq and RNA-Seq Analysis

**DOI:** 10.3390/ani15111590

**Published:** 2025-05-29

**Authors:** Liyao Bai, Jiahao Wu, Tengfei Dou, Donghui Chu, Xinjian Li, Xuelei Han, Ruimin Qiao, Kejun Wang, Feng Yang, Xiuling Li

**Affiliations:** 1 College of Animal Science and Technology, Henan Agricultural University, Zhengzhou 450046, China; b19949294189@163.com (L.B.); m17630413022@163.com (J.W.); 18567251356@163.com (T.D.); dk4cdh@163.com (D.C.); hxl014@126.com (X.H.); qrm480@163.com (R.Q.); wangkejun.me@163.com (K.W.); chinafy6868@163.com (F.Y.); 2 Henan Xunxian Pig Science and Technology Backyard, Hebi 456250, China; 3 Sanya Institute, Hainan Academy of Agricultural Science, Sanya 572025, China; lxjlongfei@163.com

**Keywords:** extrachromosomal circular DNA, circle-seq, differentially expressed genes, meat quality, pigs

## Abstract

Yunong Black (YN) pigs and Yunong Black × Landrace (YL) hybrid pigs exhibit significant differences in meat quality characteristics. Studies have suggested that extrachromosomal circular DNA (eccDNA) may play a regulatory role in muscle development. In order to study the differences in eccDNA between two groups with different meat quality traits and their potential biological significance, this study used the Circle-seq method to detect eccDNA in the longest dorsal muscle (LDM) of Yunong Black pigs (YN) (*n* = 3) and Yunong Black × Landrace hybrid pigs (YL) (*n* = 3). EccDNA-related differentially expressed genes (eccDEGs) were then analyzed in combination with RNA-seq to explore the mechanisms by which eccDNA affects meat quality. The results showed that 1325 and 1304 differentially expressed eccDNAs were identified in the YN and YL groups, varying in size and distributed across multiple genomic functional regions. These eccDNAs were also annotated according to several protein-coding genes. Combined analysis with RNA-seq results revealed 19 and 27 eccDEGs in the YN and YL groups. The Kyoto Encyclopedia of Genes and Genomes (KEGG) and Gene Ontology (GO) analysis enriched many lipid-related pathways, such as chemokine signals and ADP metabolic processes. By constructing a regulatory network, several potential regulatory networks that might be related to pork quality, for example, ecc_sus_8665/ssc-miR-212/ADAMTS16, were identified. In summary, we identified several potential eccDNAs that may regulate pig muscle, offering insights into the regulation of pig muscle traits for breeding.

## Simple Summary

The rising global demand for high-quality animal protein underscores the need for sustainable advancements in pork production. While meat quality traits are influenced by multiple genetic factors, their molecular regulatory mechanisms remain incompletely understood. Emerging evidence indicates that extrachromosomal circular DNA (eccDNA)—double-stranded circular DNA derived from chromosomal DNA—participates in muscle biology regulation, yet its functional relevance in swine remains uncharted. Here, we performed the first comprehensive analysis of eccDNA profiles in porcine longest dorsal muscle using Circle-seq technology, comparing two pig populations. Our preliminary analyses revealed significant differences in eccDNA abundance, size distribution patterns, and genomic locations. Through integrated RNA-seq analysis, we identified eccDNA-associated differentially expressed genes (eccDEGs) enriched in lipid metabolism pathways and uncovered potential regulatory networks linked to pork quality traits. These findings provide novel insights into the eccDNA-mediated genetic regulation of muscle development and offer new targets for breeding optimization.

## 1. Introduction

Pork is a major source of nutrition for humans and dominates meat production in many countries around the world. The key indicators for measuring meat quality include intramuscular fat (IMF) content, meat color, protein content, pH, marbling, and more. Pork quality is significantly influenced by factors such as feed, hygiene, care, and management practices. The balance of nutrition, the health of the pigs, and the effectiveness of management practices, including sanitation, directly impact the development of muscle quality and fat deposition in pigs. Otherwise, pork quality traits are regulated by multiple genes and are quantitative traits, which are studied using molecular genetic methods as an effective strategy for improving them [1]. In recent years, significant progress has been made in the study of pork texture traits, with different pig breeds exhibiting varying patterns of muscle and fat deposition. A genome-wide association study of 1028 pigs from six different breeds to investigate the correlation between phenotypes and traits found significant correlations between the glandular characteristics of breeds and pork quality traits (pH, meat color, fat, and moisture content) [2].

Studies have shown that transcriptomics can reveal the expression patterns of key genes during muscle growth and development, providing insights into the regulatory mechanisms involved. Currently, there are many studies on transcriptome analysis of porcine IMF, such as those using RNA-seq technology combined with lipidomics, genomics, and metagenomics approaches on the longissimus dorsi muscle (LDM) of Lantang and Large White pigs. These studies have revealed that the expression of the PLA2G12B gene regulates the production and secretion of lipoproteins [3]. Researchers have also utilized transcriptomic-assisted quantitative proteomics analysis to uncover candidate protein molecules influencing IMF deposition in Laiwu pigs [4]. Investigating the specific genes and pathways associated with IMF deposition in pigs would be of great help for breeding selection. However, a comprehensive understanding of the precise genes and regulation mechanisms responsible for the enhanced IMF deposition in animals remains limited.

Because pigs share similar physiological, pathological, and genomic characteristics with humans, recent studies have shown that human skeletal muscles of sedentary and exercising individuals have unique eccDNAs [5]. Therefore, pig skeletal muscle may also contain eccDNAs that affect traits. EccDNA refers to double-stranded circular DNA molecules originating from the genomic DNA, existing outside of chromosomes, mitochondria, and chloroplasts. Since its initial discovery in wheat embryos in 1965 [6], eccDNA has been found in a variety of organisms. It has been found in numerous eukaryotic cell types, including skeletal muscle cells from sedentary and regularly exercising individuals, in the blood and pectoral muscles of different species of pigeons, and others [5]. More and more studies have shown that eccDNA plays an important role in living organisms. For example, eccDNA serves as a gene carrier to enhance the transcription efficiency of specific genes when transferred into particular cells [7]. The first discovery of eccDNAs in pigs was observed in spermatozoa using electron microscopy [6]. Then again, it mentioned the prevalence of ecDNAs and eccDNAs detected in Weiss and Large White pigs and their ability to carry one or several partial or complete genes [8]. These findings underscore the significance of studying eccDNA in livestock and poultry.

Although eccDNAs are ubiquitous across organisms, their genetic function in pigs has been poorly studied. Given the regulatory role of eccDNAs in gene expression and their presence in pig tissues, we hypothesize that eccDNAs are involved in the genetic regulation of pork quality traits in a specific way, especially IMF deposition. To gain a deeper understanding of the genetic basis of pork quality traits, we investigated and compared the characteristics of eccDNAs in the LDM of Yunong Black (YN) pigs and YN × Landrace (YL) hybrid pigs by integrating eccDNA sequencing and transcriptome sequencing technologies. The aim was to characterize the eccDNA profiles of porcine skeletal muscle and to identify differences in eccDNA and its potential association with meat quality traits. This preliminary exploration of eccDNA characterization in pig LDM provides a new perspective for pig breeding selection.

## 2. Materials and Methods

### 2.1. Sample Description

In this study, 3 YN pigs and 3 YL pigs, with an average live weight of 106 ± 6.05 kg, were randomly selected from Henan Yifa Animal Husbandry Co., Ltd. (Hebi, China). YN pig is a new strain bred obtained by crossing the Nanyang Black Pig, Laiwu Black Pig, Erhualian Pig, and Duroc Pig [9]. All animal care and experimental procedures were conducted in accordance with the guidelines established by the Institutional Animal Care and Use Committee (IACUC) of Henan Agricultural University, China, ensuring proper welfare, housing, and management throughout the study. Both groups of pigs underwent electrical stunning and slaughter. Fresh muscle samples from the LDM at the thoracolumbar junction were collected on-site for meat quality index measurement, while another portion was immediately frozen at low temperature and transported to the laboratory for storage at −4 °C for subsequent assessment of meat quality traits. Additionally, a portion of the samples was preserved at −80 °C for testing.

### 2.2. Determination of Meat Quality Traits

The pork quality traits were determined according to NY/T 821-2019 [10], GB 5009.3-2016 [11], and GB 5009.5-2016 [12] standards. Immediately after slaughter, muscle samples measuring 2–3 cm in thickness were evaluated by the same assessor for color and marbling scores on the cross-section. The pH of the muscles at 45 min post-slaughter was measured using a calibrated pH meter (model: testo 206, Beijing Xiangruilai Technology Co., Ltd., Beijing, China) inserted into the LDM. Each sample was measured 3 times, and the average was taken. The device was calibrated using 2 buffers: 4.00 and 7.00. Additionally, the peripheral sarcolemma of some muscles was removed from the meat, and the samples were trimmed into 2 cm × 2 cm × 2 cm samples along the muscle fiber direction of the meat to detect the drip loss.

Samples of 2–10 g were placed in a clean aluminum box of constant weight and placed in a drying oven at 101–105 °C for 2–4 h to constant weight. The mass difference before and after measurement was determined, and the moisture content was calculated. Then, 1.8 g of the powder sample was placed on a constant weight neutral filter paper, wrapped, and placed in an oven at 105 °C for 2 h. Then, it was taken out and put into a Soxhlet extractor, adding 60–100 mL of anhydrous ether, heated and extracted on a water bath at 60–75 °C for 6–8 h, and placed in an oven at 105 °C for 1 h to calculate the intramuscular fat content. Another 0.2–0.5 g of the sample was placed into digestion tubes along with 3.2 g of a mixed catalyst (potassium sulfate/sodium sulfate = 1:15) and 10 mL of sulfuric acid. The samples were digested in a muffle furnace (model: SH220F, Haineng Future Technology Group Co., Ltd., Jinan, China) at 280 °C for 30 min, followed by 420 °C for 2 h. After cooling, the protein content of the muscle was determined using a Kjeldahl nitrogen analyzer (model: K9860, Haineng Future Technology Group Co., Ltd., Jinan, China).

The differences in meat quality traits between the YN and YL groups were compared using the independent samples T-test method in SPSS (v 26.0.0). This involved comparing individual meat color, tenderness, pH, drip loss, moisture content, and IMF content differences between the two groups.

### 2.3. Circle-Seq Data Analysis

For each of the 6 pigs (3 YN purebred and 3 YL hybrid), the LDM samples were individually processed and sequenced according to the Circle-seq procedure (Jiayin Biotechnology Ltd. (Shanghai, China)). It consists of the following steps: Genomic DNA was extracted using Qiagen kits (QIAGEN, Hilden, Germany). EccDNAs were enriched using the Plasmid Mini AX kit (A&A Biotechnology, Gdynia, Poland). Linear DNA was removed by Plasmid-Safe ATP-dependent DNase (Epicentre, Jiangsu, China) and digested at 37 °C for 6 days. The eccDNAs-enriched samples were used as phi29 polymerase reaction template (REPLI-g Midi Kit, QIAGEN, Hilden, Germany) to amplify DNA, which was then washed with AMPure XP beads and sonicated by Bioruptor to an average fragment size of 200 to 300 bp. Next-generation sequencing libraries were prepared using the NEBNext Ultra DNA Library Kit for Illumina according to the manufacturer’s protocol (New England Biolabs, Ipswich, MA, USA) and sequenced on an Illumina Novaseq 6000 using a PE150. Trimmomatic was used to filter the sequencing data, and parameters such as Q20 and Q30 were used to represent base quality [13]. BWA was used to compare the obtained data to the reference genome (https://ftp.ncbi.nlm.nih.gov/gnomes/all/GCF/000/003/025/GCF_000003025.6_Sscrofa11.1/, accessed on 4 March 2024) to check whether the data quality met the standard [14].

Finally, circular DNA identification was performed by matching the sequence to the reference genome through Circle-Map [15].

### 2.4. EccDNAs Difference Analysis and Gene Annotation

The eccDNAs identified through Circle-Map were processed as follows. The HOMER’s findMotifsGenome.pl tool (v 4.10.1) was used for Motif analysis. Initially, bedtools (v 2.29.2) software [16] was employed to calculate the total sum of soft-clipped read counts for each eccDNA’s start and end positions. Subsequently, DESeq2 (v 2.16.0) [17] was utilized to compute the inter-group differential expression of eccDNAs, and differentially expressed eccDNAs were selected based on predefined thresholds. The threshold criteria for this analysis were set as |log2FC| > 2 and *p* < 0.05. Functional region coordinates were extracted from the annotation file (gtf), and deepTools (v 3.4.3) were used to quantify the differential eccDNAs in these regions [18]. The distribution of eccDNAs on chromosomes and guanine-cytosine (GC) content was analyzed using the RIdeogram R package (v 0.2.2) [19]. Additionally, custom scripts and bedtools were employed for gene annotation of differential eccDNAs [16], retaining only protein-coding genes. Gene annotation analysis of differentially expressed eccDNAs was conducted using bar charts.

### 2.5. Verification of eccDNAs

To verify the accuracy of the Circle-seq results, 3 randomly selected eccDNA sequences were used to design primers and amplify different products of the expected size, which were then verified by Sanger sequencing. Primer information is provided in Table 1. Standard PCR was performed using 5xAmpliMix, with a reaction volume of 50 μL consisting of 35 μL of DNA (120 ng), 2.5 μL of forward primer (10 μM), 2.5 μL of reverse primer (10 μM), and 10 μL of 5xAmpliMix. The cycling conditions were as follows: 72 °C for 3 min, 98 °C for 30 s, 35 cycles (98 °C for 15 s, 60 °C for 10 s, and 72 °C for 8 s), 72 °C for 2 min, and storage at 10 °C. PCR products were analyzed using agarose gel electrophoresis and subjected to Sanger sequencing. Circular validation results were generated using SnapGene (https://www.snapgene.com/, accessed on 2 April 2024).

### 2.6. RNA-Seq Data Analysis

Samples stored at −80 °C were retrieved and sent for analysis (the sample information is the same as “Section 2.3. Circle-Seq Data Analysis”). RNA integrity was accurately assessed using the Agilent 2100 bioanalyzer (Agilent, Santa Clara, CA, USA). Library construction was performed following the standard NEB protocol [20]. After library construction, initial quantification was carried out using the Qubit 2.0 Fluorometer (Invitrogen, Carlsbad, CA, USA). Upon meeting the expected concentration, qRT-PCR was performed to accurately quantify the effective concentration of the library (with an effective concentration above 1.5 nM) to ensure library quality. Once the library passed quality control, Illumina sequencing was conducted, generating 150 bp paired-end reads.

Data quality control primarily involved removing adapter-containing reads, reads containing N (indicating undetermined base information), and low-quality reads (reads with more than 50% of bases with Qphred ≤ 5) [21]. HISAT2 (v 2.0.5) software [22] was employed for rapid and accurate alignment of Clean Reads to the reference genome. StringTie (v 2.2.3) software [23] performs prediction and annotation of new transcripts, while the featureCounts tool [24] in the subread (v 1.5.0) software was utilized for gene quantification analysis. Gene expression levels were represented using fragments per kilobase of transcript per million fragments mapped (FPKM). Differential expression analysis between the two comparison combinations was performed using DESeq2 (v 3.21) software, retaining only protein-coding genes. The criteria for selecting differentially expressed genes (DEGs) were |log2FC| > 1 and *p* < 0.05. Volcano plots and cluster heatmaps were generated using the NovoMagic cloud platform (https://magic.novogene.com/customer/main#/homeNew, accessed on 6 May 2024).

### 2.7. Validation of DEGs

To verify the accuracy of transcriptome sequencing results, 6 genes (UBA7, GPCPD1, AKP1C4, FRAS1, GRM4, OLFM2) were randomly selected in this study and verified by qPCR. Primers for the genes were designed on NCBI (Table 2) and synthesized by Shanghai Sangon Biotech Co., Ltd. (Shanghai, China). Tissue RNA from the two groups of individuals was extracted using the TransZol reagent kit (Zhengzhou Far East Biotechnology Co., Ltd., Zhengzhou, China), and RNA was reverse transcribed into cDNA using the Evo M-MLV reverse transcription kit (Hangzhou Aikerui Biotechnology Co., Ltd., Hangzhou, China). qPCR validation was performed using the SYBR Green I (Hangzhou Aikerui Biotechnology Co., Ltd., Hangzhou, China) intercalation fluorescence method with GAPDH as an internal reference. The reaction system consisted of 10 μL: 5 μL 2× SYBR, 1 μL cDNA, 0.2 μL each of upstream and downstream primers, and 3.6 μL RNase-free water. The gene expression was calculated by 2^−ΔΔCt^.

### 2.8. Enrichment Analysis and Functional Annotation of eccDNAs and eccDEGs

To further uncover the molecular mechanism of eccDNAs and eccDEGs, enrichment analysis was performed based on the results of the two-omics combination to determine candidate eccDEGs related to meat quality traits. Specifically, functional enrichment analysis of genes was performed using a free online data analysis platform (https://www.omicshare.com/tools accessed on 18 June 2024). KEGG and GO pathway enrichment analyses were conducted using gene IDs on the Novogene cloud platform (https://magic.novogene.com/customer/main#/homeNew accessed on 18 June 2024), and graphical representations were generated through the website (https://www.bioinformatics.com.cn/, accessed on 10 July 2024).

### 2.9. Prediction of Regulatory Network of eccDNAs-miRNAs-Genes

Porcine miRNA sequences were extracted from the miRBase database (http://www.mirbase.org/, accessed on 15 August 2024), and the 3′UTR sequences of the genes were retrieved from UCSC (https://genome.ucsc.edu/, accessed on 15 August 2024). The miRanda (v.3.3a) software (https://www.bioinformatics.com.cn/local_miranda_miRNA_target_prediction_120, accessed on 2 June 2024) was used to predict pig miRNAs that might interact with eccDNAs and their target genes. The prediction was performed using the default parameters of the system. Cytoscape (v.3.10.1) was used for visualization.

## 3. Results

### 3.1. Comparison of Meat Quality Indicators in the YN and YL Groups

The results of LDM quality determination for both groups of pigs (Table 3) indicate that several meat quality indicators of YN pigs are significantly higher than those of YL pigs. The meat color score assessed 45 min post-slaughter was significantly higher in YN pigs compared to YL pigs (*p* < 0.05). There was also a significant difference between the two groups in terms of pH (*p* < 0.05). Specifically, the intramuscular fat content of the longest dorsal muscle in YN pigs was significantly higher than that in YL pigs (*p* < 0.01).

### 3.2. Basic Features of eccDNAs in YN and YL Pigs

To characterize eccDNAs in YN and YL pigs, Circle-seq was employed for high-throughput sequencing of eccDNAs, and the raw sequencing data were filtered. After filtering, the overall quality of the raw reads was evaluated using Q30 as the QC standard. A Q30 > 80% indicates good sequencing quality. The results showed that the mean Q30 values of individuals in the YN and YL groups were both greater than 93%, and 87,020 and 78,439 eccDNAs were identified in the two groups, respectively (Supplementary Table S1).

**Figure 1:**
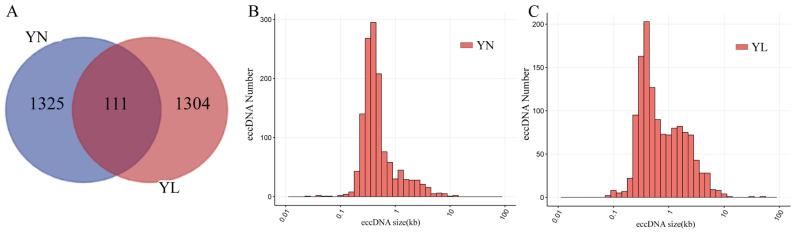
eccDNA sequencing results. (**A**) Venn diagram illustrating differential eccDNAs. (**B**,**C**) Distribution of differential eccDNA lengths.

There were 1325 and 1304 different eccDNAs in the YN and YL groups, respectively (Figure 1A). The lengths of differential eccDNAs also differ between the two groups in this study (Figure 1B,C). The length of differential eccDNAs in the YN and YL groups was mainly distributed between 0.1–10 kb, while that in the YN group was concentrated between 0.1 and 1 kb. However, the length of differential eccDNAs in the YL group was between 1–10 kb more than that in the YN group. Longer eccDNAs can carry longer functional genome segments.

**Figure 2:**
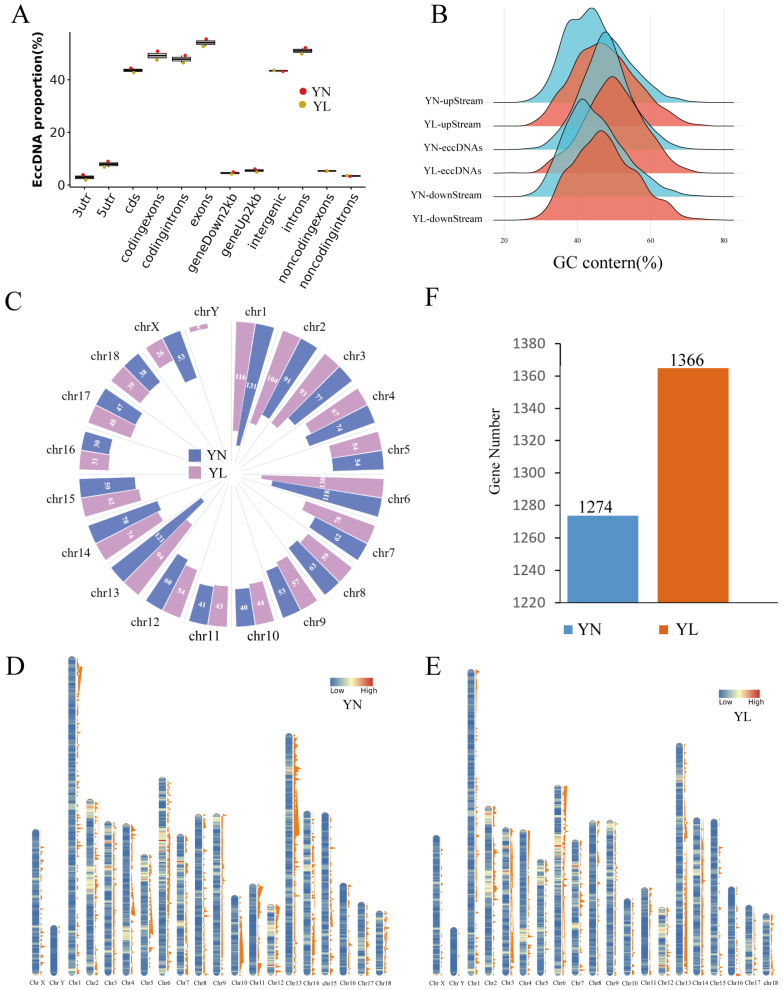
Chromosomal distribution of eccDNAs and gene annotation of eccDNAs. (**A**) Proportional representation of differential eccDNAs across genomic functional regions. (**B**) The analysis of the GC content of differential eccDNAs and their upstream and downstream regions with equivalent lengths (1000 bp) from the YN and YL groups. Downstream: represents the downstream region of eccDNA; eccDNA: represents the entire coordinate interval of eccDNA; upstream: represents the upstream region of eccDNA. (**C**) Differences between the YN and YL groups eccDNA distribution numbers on chromosomes. (**D**) The chromosomal distribution of differential eccDNAs in the YN group. (**E**) The chromosomal distribution of differential eccDNAs in the YL group. The depth of chromosome color represents gene density, while varying shades of orange indicate the number of differential eccDNAs on the chromosome, with higher shades indicating a greater abundance of differential eccDNAs. (**F**) The number of annotated genes associated with differential eccDNAs.

To understand the impact of eccDNA on coding and regulation, the genomic element distribution was analyzed (Figure 2A). EccDNAs from the two groups showed a similar genomic element distribution pattern. The results indicate that differential eccDNAs in both groups are localized to different categories of genomic regions, primarily originating from coding sequence (CDS), coding exons, coding introns, and intergenic regions. The proportion of differential eccDNAs in genomic functional regions is slightly higher in the YL group compared to the YN group. For example, in the exons region, the eccDNA proportion of the YN group is 55.44%, and that of the YL group is 52.68%; in the CDS region, the eccDNA of the YN group accounted for 44.40%, and that of the YL group accounted for 42.72%, which may indicate a greater coding capacity. The GC content of differential eccDNA in the YN and YL groups was measured at 47.82% and 49.82%, respectively, both surpassing the GC content detected in the upstream flanking regions (44.88% and 47.47%) or downstream flanking regions (44.88% and 47.53%) of the identified differential eccDNA loci, indicating the possible transcriptional-translational capacity of eccDNAs in the LDM (Figure 2B).

The differential eccDNAs in both the YN and YL groups were predominantly distributed on chromosomes 1, 6, and 13, but there were differences in the distribution of specific locations on the chromosomes (Figure 2C–E). In particular, on chromosome 13, the YN group exhibits a significantly higher number of differential eccDNAs than the YL group (121 differential eccDNAs in the YN group versus 94 in the YL group). To further investigate the mechanisms of action of these differential eccDNAs, we annotated genes associated with them and identified protein-coding genes in both groups. There are 1274 and 1366 genes identified in the YN and YL groups, respectively (Figure 2F).

### 3.3. Validation of eccDNAs

**Figure 3:**
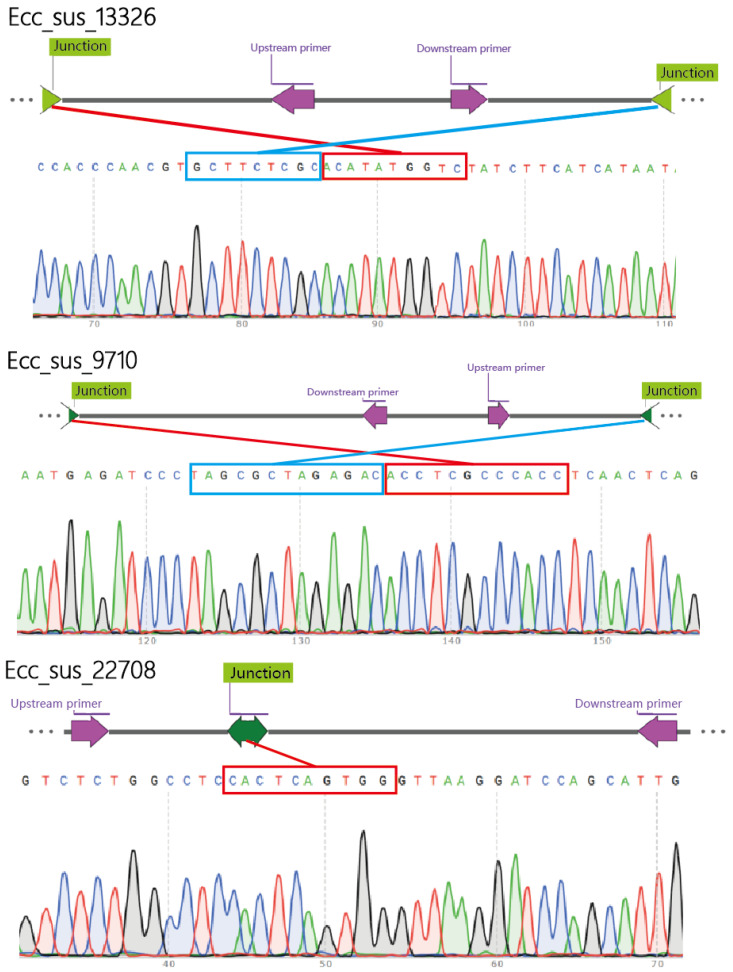
Circular validation result of the eccDNAs. Upstream primer: represents the upstream primer location for circular validation PCR (the green arrow indicates); Downstream primer: represents the downstream primer location for circular validation PCR (the green arrow indicates); junction: represents the circular link site (the purple arrow indicates).

Circular validation of eccDNA was conducted by randomly selecting ecc_sus_13326, ecc_sus_9710, and ecc_sus_22708. Specific primers were designed to amplify sequences containing cleavage sites via PCR, followed by the validation of PCR products using Sanger sequencing (Table 1). The results demonstrate that the cleavage sites of these three eccDNAs were successfully amplified, which is consistent with our Circle-seq results (Figure 3).

### 3.4. Identification of DEGs

**Figure 4:**
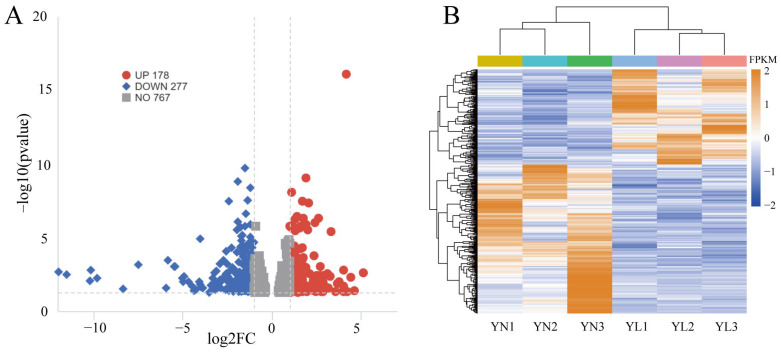
Transcriptome sequencing results. (**A**) Volcano plot of DEGs (up: genes expression was higher in the YN group than in the YL group, down: genes expression was lower in the YN group than in the YL group). (**B**) Heatmap of clustered DEGs.

The relevant results of RNA-seq are as follows: a total of 455 differentially expressed protein-coding genes were identified (Figure 4A, Supplementary Table S2). Clustering analysis based on gene FPKM values is shown, with blue representing low-expression genes and orange representing high-expression genes. A total of 178 up and 277 down genes (|log2 FC| > 1, *p* < 0.05) were found. Significant differences in gene expression levels among groups were observed (Figure 4B).

The six DEGs were randomly selected from the transcriptome for qPCR validation, with primer information listed in Table 2. The qPCR results (Supplementary Figure S1A) demonstrate consistent trends in expression levels of up-regulated genes UBA7, GPCPD1, and AKP1C4, and down-regulated genes FRAS1, GRM4, and PER2 were similar to those of the RNA-seq results (Supplementary Figure S1B), validating the confidence of the sequencing results, with significant differences observed in expression levels between the two groups.

### 3.5. Identification and Enrichment Analysis of Differentially Expressed eccDNA-Amplified Encoding Genes

Combined with the transcript expression level of DEGs, the overlapped parts between differentially expressed eccDNA genes and DEGs, called eccDNA-related DEGs (eccDEGs), were further studied. In all, there are 19 eccDEGs (Supplementary Table S4) in the YN group and 23 eccDEGs (Supplementary Table S5) in the YL group. The full names of the genes listed in Supplementary Tables S4 and S5 can be found in Supplementary Table S3.

**Figure 5:**
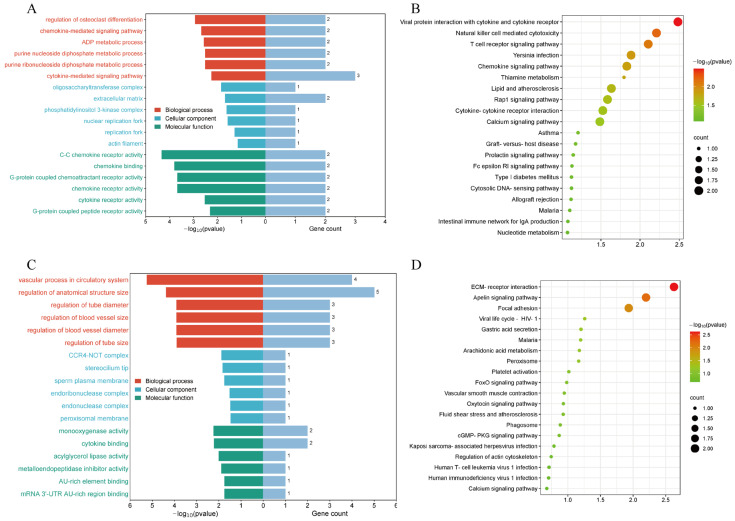
Enrichment analysis of eccDEGs. (**A**,**B**) GO and KEGG enrichment analysis of binding eccDEGs in the YN group. The colors in the GO results represent different categories: Red indicates Biological Process (BP), Blue represents Cellular Component (CC), and Green corresponds to Molecular Function (MF). (**C**,**D**) GO and KEGG enrichment analysis of binding eccDEGs in the YL group.

The KEGG and GO enrichment analysis results of the two groups of eccDEGs are shown in Figure 5. Pathways enriched in the YN group include viral protein interaction with cytokine and cytokine receptors, chemokine signaling pathway, and lipid and atherosclerosis (Figure 5B). Significantly enriched GO terms in the biological processes (BPs) include the ADP metabolic process, the purine nucleoside diphosphate metabolic process, and the cytokine-mediated signaling pathway (Figure 5A). Pathways enriched in the YL group include the Apelin signaling pathway, arachidonic acid metabolism, and the FoxO signaling pathway (Figure 5D). Significant GO terms in the BPs include the vascular process in the circulatory system and the regulation of tube diameter (Figure 5C).

### 3.6. EccDNAs–miRNAs–Genes Regulatory Network

**Figure 6:**
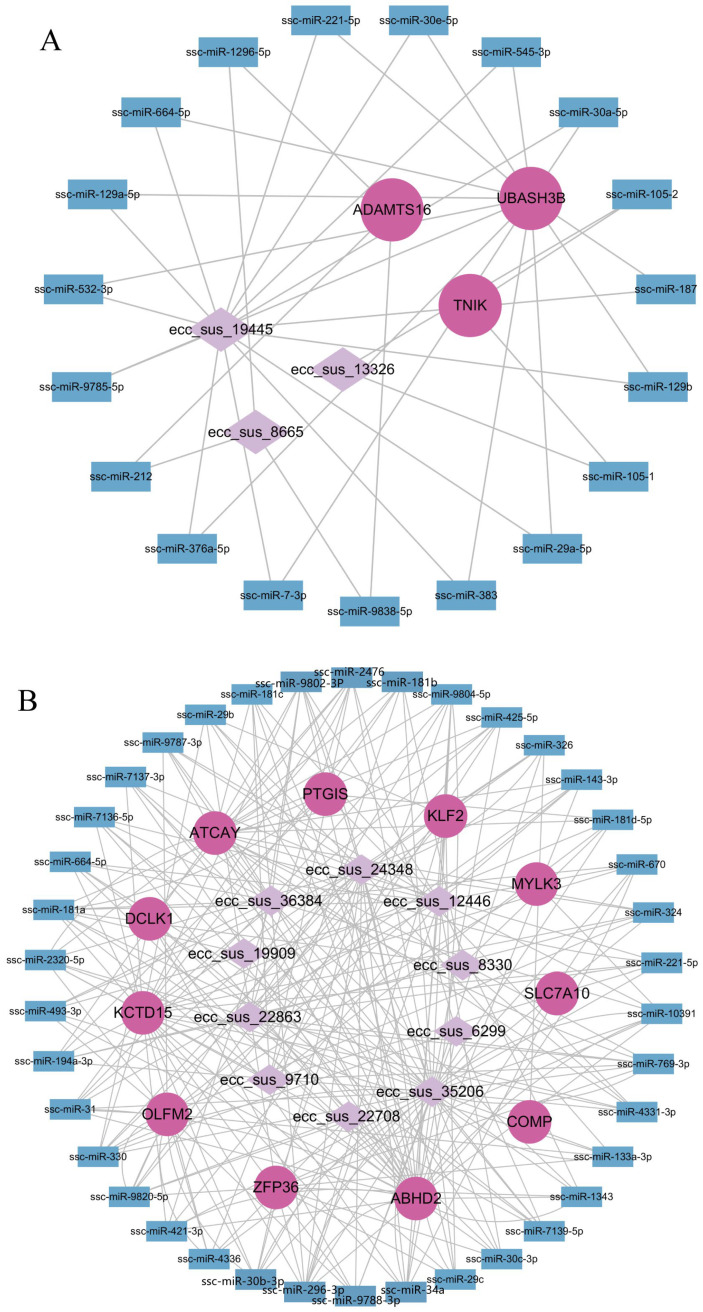
Construction of the eccDNAs–miRNAs–genes regulatory network. (**A**) Analysis of the eccDNAs–miRNAs–genes network in the YN group; (**B**) Analysis of the eccDNAs–miRNAs–genes network in the YL group. Diamond nodes indicate eccDNAs, circular nodes indicate genes, and square nodes indicate mRNAs.

Increasing evidence suggests that eccDNAs may function as molecular sponges, typically playing a regulatory role in various biological processes. Therefore, we hypothesize that the eccDNAs in this study might act as miRNA sponge regulatory genes. The results from miRanda predictions revealed that the differing eccDNAs between the YN and YL groups were associated with multiple miRNAs, for example, ssc-miR-212, ssc-miR-432-5p, and ssc-miR-1343 (Figure 6). Each eccDNA can interact with multiple miRNAs, such as ecc_sus_8865, ecc_sus_22708, and ecc_sus_9710, and each miRNA can also be associated with multiple eccDNAs. Additionally, the miRNAs predicted by the eccDNAs also interact with eccDEGs, such as ADAMTS16, OLFM2, and PTGIS, which are involved with multiple miRNAs. Multiple regulatory networks, for instance, ecc_sus_8665/ssc_miR_212/ADAMTS16 and ecc_sus_22708/ssc-miR-432-5p/OLFM2 were found. Therefore, the eccDNAs in this study may influence traits by regulating the expression of related genes through miRNAs.

## 4. Discussion

In this study, we integrated eccDNA sequencing and transcriptome sequencing data from six samples (three from the YN group and three from the YL group) to explore the differences in eccDNA in the LDM in YN and YL pigs and their potential biological functions. Finally, 1325 and 1304 differential eccDNAs were detected in the YN and YL groups. We compared the differential eccDNA quantities, length distributions, genomic functional region distributions, chromosomal distributions, and differences in the functional genes of differential eccDNAs between the two pig groups. This analysis aimed to reveal the differences in eccDNA in pigs with different meat quality traits and the potential biological significance of this.

In the pig industry, meat quality is one of the most important economic traits globally, and it is influenced by multiple factors, including heredity and the environment [25]. It is well-known that indigenous pigs exhibit better meat quality compared to commercial lean pigs. The results indicate that YN pigs exhibit superior meat quality compared to YL pigs, as evidenced by higher meat color scores, lower pH, and higher intramuscular fat content. This is consistent with previous research on local breeds of pigs, such as the Songliao Black Pig, which shows higher backfat, intramuscular fat, and pH24 compared to the commercial crossbreed of Large White × Landrace [26]. Muscle development is a complex biological process that is regulated by multiple mechanisms. Studies of Circle-seq and RNA-seq in human skeletal muscle provide a preliminary understanding of the regulatory network of eccDNAs in skeletal muscle development [5].

Since the discovery of eccDNA, researchers have employed next-generation sequencing technologies and data analysis methods to reveal its structural features. Firstly, eccDNA exhibits a wide range of sizes, from tens of base pairs to several million base pairs. In this study, differential eccDNA sizes identified in YN and YL pigs using Circle-Seq technology ranged from tens of base pairs to tens of kilobases, with the majority of differential eccDNA lengths showing a micro-homogeneity between the two groups. This finding is consistent with numerous previous studies. The eccDNA in cattle was distributed across autosomes, covering 4.6% of their length, with an average size of 33,474.9 bp, predominantly ranging from 50 to 6000 bp [27]. The regions forming eccDNAs are not randomly distributed across chromosomes. In this study, differential eccDNAs were found on all chromosomes, although their density was higher on certain chromosomes, which is consistent with previous studies. Overall, many factors influence the size distribution of eccDNA, such as different ages and breeds [28]. Therefore, eccDNA length may contribute to phenotypic differences among different breeds.

Studies have shown that 33 to 54,229 bp eccDNA can play a role as a gene regulatory element affecting traits [29]. In this study, two groups of differential eccDNAs were localized to distinct genomic regions, primarily originating from CDS regions and protein-coding exon sequences. Multiple eccDNAs and ecDNAs were annotated to exons, introns, and gene regions in both Wei pigs and Large White pigs [8]. Differential eccDNAs in both the YN and YL groups in this study were found more frequently in regions encoding genes, which may contribute to elevated transcription levels, suggesting that eccDNA in specific genomic regions may have potential physiological impacts. Furthermore, the GC content of differential eccDNAs was similar between the two groups, consistent with previous findings in plasma [30], femoral head [31], and adipose stem cells [32]. However, their GC content was higher than that of flanking sequences, suggesting that the identified differential eccDNAs may have more stable transcriptional and translational capabilities.

In recent years, the biological function of eccDNA has gained increasing attention, particularly its important role in diseases such as cancer, genomic instability, and drug resistance. By annotating the target genes of eccDNA, we can uncover their underlying functions and mechanisms. For example, eccDNA regulates the white dorsal stripe trait in cattle through two chromosomal translocations involving the KIT gene, resulting in the formation of Cs29 on chromosome 29 and Cs6 on chromosome 6 [33]. Therefore, eccDNAs in this study may also influence pork quality traits through their associated genes. We annotated the genes of the differential eccDNAs in the two groups and integrated them with transcriptome data to identify the eccDEGs carried by the differential eccDNAs in the YN and YL groups. Enrichment analysis of the KEGG pathways in the YN group showed that the eccDEGs were enriched in the chemokine signaling pathway, lipid metabolism, and the atherosclerosis pathway. Studies have demonstrated that experimental atherosclerosis in rats is associated with lipid metabolism [34]. The significant GO terms include the ADP metabolic process, among others. EccDNA also plays important biological roles by regulating signaling pathways. Sun et al. [35] found that the stability of DMs in tumor cells is related to the activation of ERK1/2. The inhibition of ERK1/2 activation and constitutive phosphorylation of ERK1/2 significantly reduced the number of DMs in tumor cells and the expression of DM-carrying genes. The KEGG enrichment pathways in the YL group included the arachidonic acid metabolism, among others. Scholars have identified, through proteomic and metabolomic analyses, that the arachidonic acid metabolic pathway may be associated with lipid metabolism in male mice [36]. The significant GO terms for BP enrichment included vascular processes in the circulatory system and tube diameter regulation, among others. The GO terms and KEGG pathways enriched in both groups were similar, likely because the experimental animals in both groups were YN pigs. The analysis of GO and KEGG pathways derived from the eccDEGs annotation revealed the involved cell signaling pathways and the underlying biological mechanisms.

Previous studies have utilized artificially synthesized eccDNA carrying H3K27ac-modified genomic regions and introduced them into cells to assess their impact on global gene expression, investigating whether they affect transcription [37]. In this study, to identify eccDNAs worthy of further investigation, we selected eccDEGs related to meat quality traits and identified their corresponding eccDNAs as the next focus of research. The results revealed that in the YN group, the ADAMTS16 gene influences muscle development by interacting with LAP-TGF-β signaling [38]. In the YL group, eccDEGs such as KLF2, OLFM2, and COMP are associated with white adipocyte differentiation [39] and protein determination [40]. Although the direct link between differences in eccDNA and meat quality traits has not been explicitly analyzed in this study, the differential expression of eccDEGs in the YN and YL pig groups suggests a potential relationship. Therefore, these eccDNA elements may influence the meat quality traits of YN pigs through the regulation of eccDEGs.

However, the regulation of eccDNA traits is a complex process that may involve additional regulatory factors. To further understand how eccDNA regulates genes and influences traits, we performed miRNA interaction network predictions on the final screened eccDNAs and eccDEGs. Several miRNAs with interactions were identified in both groups. Previous studies have confirmed that eccDNA can be associated with bmo-miR-3318 and regulate the expression of its target gene LOC101742450 through miRanda predictions and DNA pull-down experiments [41,42]. Additionally, research has shown that eccDNA-carrying miRNA genes can produce functional miRNA molecules in host cells, promoting cell growth and survival [43]. This supports the hypothesis that eccDNAs may regulate the expression of genes through miRNA-mediated pathways, influencing the phenotype related to meat quality. Based on these findings, we hypothesize that the differential eccDNAs in this study may also regulate the expression of eccDEGs by interacting with miRNAs, thereby influencing meat quality-related traits. The next step will involve selecting appropriate eccDNAs for experimental validation to further elucidate the mechanisms of eccDNA in both YN and YL pigs.

As with the majority of studies, the design of the current study is subject to limitations. The relatively small sample size (3 + 3) in our study is a limitation that can impact the statistical power and generalizability of the findings. Therefore, future studies with larger sample sizes would be valuable to further validate our findings and enhance their applicability to a wider range of pigs.

## 5. Conclusions

Our study investigated the characteristics of eccDNA in YN and YL pigs and found that there were differences in eccDNAs in terms of length and chromosome distribution between the two groups. We aimed to identify candidate genes that might be related to meat quality, such as ADAMTS16, TNIK, OLFM2, COMP, and PTGIS, through an integrated analysis of eccDNAs and eccDEGs. Notably, there are multiple candidate regulatory networks, such as ecc_sus_8665/ssc_miR_212/ADAMTS16 and ecc_sus_22708/ssc-miR-432-5p/OLFM2, which were identified as potentially involved in pork quality regulation. The expression patterns and correlations of these eccDNAs and genes in YN and YL samples further emphasize their potential contributions to meat quality. Although these findings provide valuable insights into the potential regulatory roles of eccDNAs in pork quality, the detailed molecular mechanisms remain unclear. Future research should focus on the functional validation of these eccDNAs and eccDEGs, as well as their precise roles in the genetic regulation of meat quality traits, using larger sample sizes to enhance the robustness of the results.

## Figures and Tables

**Table 1:** The primers used in eccDNAs verification.

eccDNAs	Forward Primers	Reverse Primers
Ecc_sus_13326	CAATCGCACCAGTGAGGCC	GGTGGTGTTGAGGATGAATGTG
Ecc_sus_9710	ATTCCCATTGTGGCTCTTG	CACAAATCCAAAGGGAACAA
Ecc_sus_22708	GAGATGTGCCAGGTGGACTGT	GTAATCAACCCTGACCATCTCCT

**Table 2:** The primers used in gene expression.

Gene Name	Forward Primers	Reverse Primers
UBA7	CTTCTGCTGAGTTTGGCCCT	ATAGTTCTGAGCTCGCAGGC
GPCPD1	TCTGGTGGGAGCTTTGCTTT	TGGTGGGGATTTGTCTTCCAA
AKR1C4	GACATCGAGGTGCAGGGAAT	CAAAGGCTGCACCGTGACTA
GRM4	GTCGGCAGACAGATGGTCTT	TGCTCTTAGGGACCAAATCCC
FRAS1	GTCAAGAAGTGCACCAACCG	CGGCATCGATCACAAACTGC
PER2	ACTTCGTCTTCCTGTCCAGATG	CTTTCAGCTCCCTCAGCGTT

**Table 3:** Meat quality characteristics of LDM in the YN and YL groups.

Item	YN	YL
Meat color 45 min	4.00 ± 0.00 ^a^	2.40 ± 0.17 ^b^
Ph 45 min	5.45 ± 0.12 ^b^	6.17 ± 0.15 ^c^
Marble grain 45 min	3.50 ± 0.50	2.50 ± 0.50
Drip loss %	1.04 ± 0.12	1.10 ± 0.27
Moisture %	73.66 ± 1.66	75.14 ± 0.66
Intramuscular fat content (dry sample) %	13.80 ± 0.63 ^a^	9.39 ± 0.44 ^c^
Protein content (dry sample) %	77.90 ± 0.87	81.57 ± 2.77

Note: The superscript letters on the data shoulder indicate significant differences (*p* < 0.05), with alternating letters indicating highly significant differences (*p* < 0.01). Rows lacking letters or having identical letters indicate non-significant differences (*p* > 0.05).

## Data Availability

The original contributions presented in the study are included in the article/Supplementary Material; further inquiries can be directed to the corresponding author.

## Supplementary Materials

The following supporting information can be downloaded at https://www.mdpi.com/article/10.3390/ani15111590/s1, Figure S1: The six DEGs for qPCR validation; Table S1: Sequencing quality of eccDNA in YN and YL groups; Table S2: Results of RNA-seq sequencing; Table S3: The full name of eccDEGs of the YN and YL groups; Table S4: Up-regulated eccDEGs in YN and YL group; Table S5: Down-regulated eccDEGs in YN and YL group.
