# Supplementary figures and images for: Initial Screening of Extrachromosomal Circular DNA Candidates for Pork Meat Quality Traits Using Circle-Seq and RNA-Seq Analysis
Source: Animals (Basel). 2025 May 29;15(11):1590. doi: 10.3390/ani15111590 (PMC12153846; doi:10.3390/ani15111590)

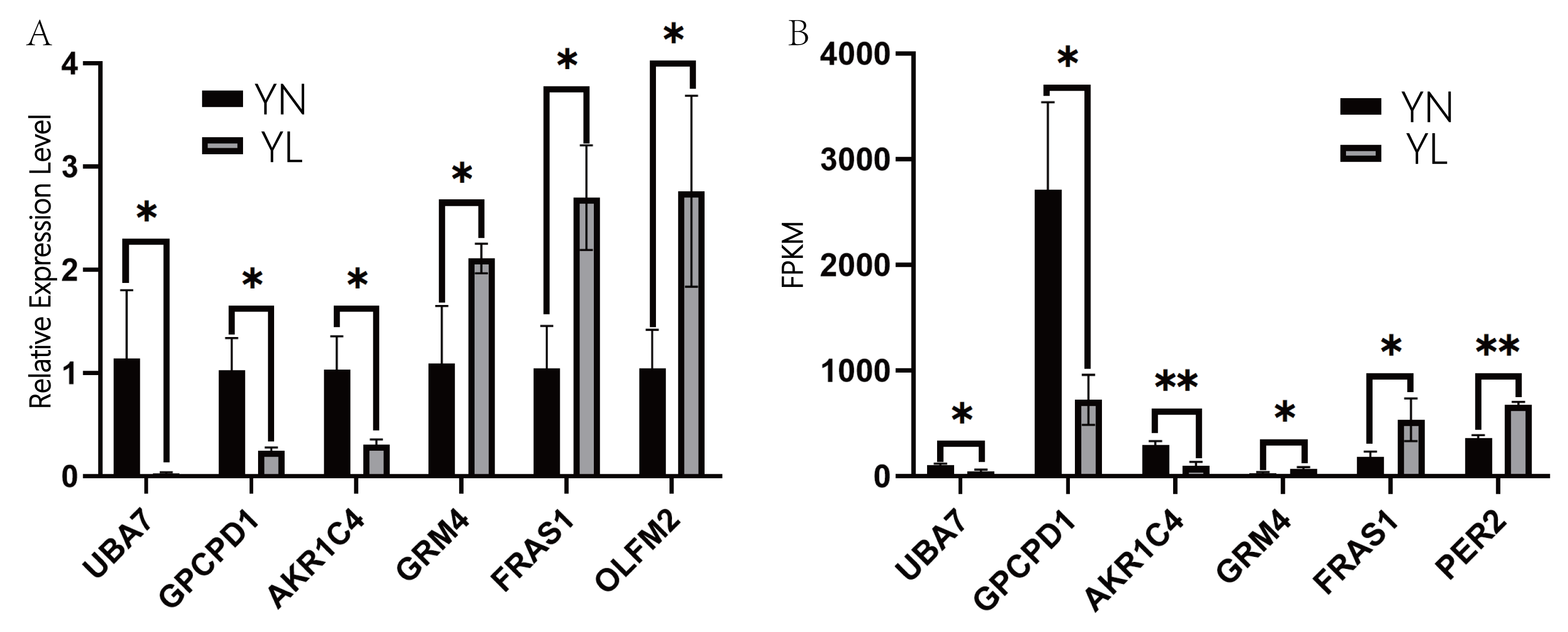

Supplement: Supplementary file 1 [file animals-15-01590-s001.zip › Supplementary Figures S1A and S1B.png]
